# Scleroderma-polymyositis overlap syndrome versus idiopathic polymyositis and systemic sclerosis: a descriptive study on clinical features and myopathology

**DOI:** 10.1186/ar4562

**Published:** 2014-05-13

**Authors:** Kavish J Bhansing, Martin Lammens, Hanneke KA Knaapen, Piet LCM van Riel, Baziel GM van Engelen, Madelon C Vonk

**Affiliations:** 1 Department of Rheumatic Diseases, Radboud Medical Center, Nijmegen, The Netherlands; 2 Department of Pathology, Antwerp University Hospital, Antwerp, Belgium; 3 Scientific Institute for Quality of Healthcare, Radboud University Medical Center, Nijmegen, The Netherlands; 4 Department of Neurology, Radboud University Medical Center, Nijmegen, The Netherlands

## Abstract

**Introduction:**

The objective was to characterize the clinical and myopathologic features of patients with scleroderma-polymyositis (SSc-PM) overlap compared with a population of patients with systemic sclerosis (SSc) and polymyositis (PM).

**Methods:**

A three-way comparison of patients with SSc-PM overlap (*n* = 25) with patients with SSc (*n* = 397) and PM (*n* = 40) on clinical and myopathologic features and causes of death. One neuropathologist blinded for the diagnosis evaluated all recent available muscle biopsies. Biopsies were scored for presence of inflammation, necrotic muscle fibers, rimmed vacuoles, fibrosis, and immunohistochemical staining. Clinical or myopathologic characteristics were compared by using the χ^2^ test or one-way analysis of variance (ANOVA).

**Results:**

The prevalence of SSc-PM overlap in the Nijmegen Systemic Sclerosis cohort was 5.9%. The mortality was 32% (eight of 25) in SSc-PM, of which half was related to cardiac diseases. The prevalence of pulmonary fibrosis was significantly increased in SSc-PM (83%) (*P* = 0.04) compared with SSc (49%) and PM (53%). SSc or myositis-specific antibodies were nearly absent in the SSc-PM group. In almost all biopsies (96%) of SSc-PM patients, necrotic muscle fibers were present, which was significantly increased compared with PM patients (*P* = 0.02).

**Conclusions:**

Patients with SSc-PM have increased prevalence of pulmonary fibrosis and cardiac disease as the cause of death compared with patients with SSc and PM . In addition, we found that necrotizing muscle fibers with inflammation characterize SSc-PM overlap in muscle biopsies. Further research should focus on underlying mechanisms causing necrosis, inflammation, and fibrosis and their relation to pulmonary involvement and mortality in patients with SSc-PM overlap.

## Introduction

Systemic sclerosis (SSc) is a systemic autoimmune disease characterized by vascular lesions and fibrosis of multiple organs, predominantly in skin, lungs, heart, intestinal tract, joints, and muscles [1,2]. The prevalence of myopathies in SSc patients varies from 5% to 81%, depending on the use of different definitions of muscle involvement [3-11]. Myositis in SSc patients resembles clinical and biologic features of patients with polymyositis (PM), hence the term scleroderma/polymyositis overlap (SSc-PM). Previous studies demonstrated a worse survival and increased prevalence of myocardial involvement in SSc-PM overlap compared with SSc [9,12]. Therefore, it seems clinically relevant to identify SSc-PM overlap for close monitoring and early treatment. To date, no studies have been conducted to characterize the full spectrum of SSc-PM overlap by means of a three-way comparison with SSc and idiopathic PM. Better understanding of similarities and differences in clinical and biologic features as well as outcome may lead to improved treatment and prognosis for SSc-PM overlap patients.

The aim of this study is to characterize the clinical and myopathologic features of patients with SSc-PM overlap patients compared with a population of patients with SSc and PM.

## Methods

### Design

The Nijmegen Systemic Sclerosis cohort is an ongoing, prospective inception cohort started in 1989 at the Department of Rheumatic Diseases at the Radboud University Medical Center. The data collection contains information of symptoms, physical examination, laboratory workup, as well as annually performed pulmonary-function test results, echocardiography, right-heart catheterization, and high-resolution computed tomography (HRCT) scans at baseline and when indicated.

The Nijmegen Myositis cohort is also a prospectively followed-up cohort of patients with inflammatory myopathies. This cohort includes all patients with inflammatory myopathies from the Computer Registry of All Myopathies and Polyneuropathies (CRAMP) treated at the Radboud University Medical Center [13]. The CRAMP is a Dutch multicenter neuromuscular registry and was developed in 2004. The data available of the Nijmegen Myositis cohort consist of demographic and clinical features at diagnosis combined with follow-up information on treatment, biochemical markers, pulmonary-function test results, and, if indicated, HRCT scans and echocardiography.

#### Participants

All patients with SSc can be classified by the ACR preliminary classification criteria for SSc or Leroy criteria for early SSc. The patients with PM all fulfilled the Bohan and Peter diagnostic criteria, whereas the SSc-PM overlap participants fulfilled both criteria [14-17].

All SSc-PM patients of the Nijmegen Systemic Sclerosis cohort were included. SSc patients with serum CK more than 2 times upper limit, myalgia, and proximal muscle weakness were analyzed for the presence of polymyositis with electromyography (EMG) and muscle biopsy. All consecutive PM patients of the Nijmegen Myositis cohort were included. The study was exempted from approval of the local Medical Ethics Committee Arnhem-Nijmegen in the Netherlands, because this was an observational, noninterventional study. Therefore, no informed consent was required for this study.

#### Myopathologic analysis

The slides of all available muscle biopsies of SSc-PM (*n* = 24) and most-recent consecutive patients with PM (*n* = 24) were used to pair with a 1:1 ratio. One neuropathologist (ML), blinded for diagnosis, evaluated all muscle biopsies. Biopsies were scored for the presence of inflammatory infiltrates, necrotic muscle fibers, fibrosis, rimmed vacuoles, and, if performed for enzyme histochemistry, for cytochrome C-oxidase (COX) and succinate dehydrogenase (SDH) and immunohistochemistry for MHC class I, membrane attack complex (MAC), CD 4, CD 8, CD 20, and CD 68. Inflammatory infiltrates were defined as the presence of mononuclear cell infiltrates surrounding or invading muscle fibers. Necrosis in muscle biopsy was defined as presence of acute necrotic muscle fibers, identified on hematoxylin–phloxine staining (paling of the cytoplasm and absence of basophilia), myophagia, or the presence of regenerating basophilic fibers. Fibrosis was defined as increase of collagen and fibroblasts in the endomysium. MHC class I upregulation was regarded as positive if at least the sarcolemma expressed MHC class I antigen [18]. MAC upregulation was regarded as present if MAC was expressed in the endothelium of capillaries or in the sarcoplasm of muscle fibers [19,20].

#### Statistical analysis

Statistical analysis was performed with SPSS version 20 for Windows (SPSS Inc., Chicago, IL, USA). Study population and muscle-biopsy characteristics were compared with the χ^2^ test or Fisher Exact test for nominal data and the Mann–Whitney *U* test for numeric data or one-way ANOVA. Statistical significance was defined as *P* ≤ 0.05.

## Results

The mean age at diagnosis was 53 years (SD, 12.3) (SSc-PM overlap), 50 years (SD, 13.2) (SSc), and 51 years (SD, 16.5) (PM). The median disease duration was 5.0 years (IQR, 2.8 to 12.4), 7 years (IQR, 3 to 14), and 3.5 years (IQR, 2 to 11), respectively. The SSc-PM group was characterized by an almost 1:1 female/male ratio, whereas the SSc group by 2:1 female-to-male ratio, and the PM group by an almost opposite ratio of 1:2 (Table 1). The prevalence of SSc-PM overlap in the Nijmegen Systemic Sclerosis cohort was 5.9%.

**Table 1:** Study population characteristics

**Characteristics**	**Number**	**SSc-overlap (*n*** **= 25)**	**Number**	**SSc (*n*** **= 397)**	**Number**	**PM (*n *** **=40)**	***P *** **value**
Age in years, mean (SD)	25	53 (12.3)	397	50 (13.2)	40	51 (16.5)	0.013
Gender (male/female)	25	12/13	397	131/266	40	25/15	0.003
Type diagnosis							
*Limited cutaneous*		19 (76%)		276 (73%)		NA	NS
*Diffuse cutaneous*		6 (24%)		109 (27%)			
Disease duration, years median (IQR)	25	5 (3–12)	397	7 (3–14)	40	3.5 (2–11)	0.001
Mortality	25	8 (32%)	397	65 (16%)	40	7 (18%)	NS
Survival in years, median (IQR)	8	2.5 (1.3-9.6)	65	7 (3–13.5)	7	0.3 (0–12)	NS
***Serology***							
ANA	25	25 (100%)	397	356 (90%)	34	21 (62%)	0.006
Anti-topoisomerase	21	0	397	87 (22%)	31	1 (3%)	0.048
Anti-centromere	19	2 (10%)	397	76 (19%)	31	0	NS
Anti-SSA	25	2 (8%)	397	15 (4%)	31	11 (36%)	< 0.001
Anti-SSB	25	0		NA	31	1 (3%)	NS
Anti-RNP	25	2 (8%)	397	27 (7%)	31	2 (6%)	NS
Anti-SM	25	0		NA	31	2 (6%)	NS
Anti-Jo1	25	2 (8%)		NA	31	13 (42%)	0.013

NA, not applicable; NS, not significant.

### Serology

SSc-specific autoantibodies (anticentromere and antitopoisomerase 1), were nearly absent in the SSc-PM group and present in 19% and 22% of the SSc patients. None of the PM patients tested positive for anticentromere and antitopoisomerase 1, except one PM patient. Myositis-associated antibody, anti-SSA and myositis-specific antibody, anti-Jo1, were significantly less present in the SSc-PM group compared with the PM groups (Table 1).

### Scleroderma and myositis-specific features

Raynaud phenomenon was present in all groups: 21 patients (84%) in SSc-PM overlap, 379 patients (96%) in SSc, and 13 patients (33%) in PM. The 13 (33%) patients with Raynaud phenomena in the PM group also had features such as mechanic hands, arthritis, ILD, and were anti-Jo1 antibodies positive, reflecting the anti-synthetase syndrome. The median modified Rodnan skincore (mRSS) was 6 (IQR, 5 to 11) in the SSc-PM group and 7 (IQR, 4 to 12) in the SSc group (Table 2).

**Table 2:** Disease-specific characteristics

**Characteristics**	**Number**	**SSc-overlap (*n*** **= 25)**	**Number**	**SSc (*n*** **= 397)**	**Number**	**PM (*n*** **= 40)**	***P *** **value**
***Scleroderma features***							
Raynaud phenomena	25	21 (84%)	397	379 (96%)	40	13 (33%)	<0.001
Digital ulcers	25	6 (24%)	397	164 (41%)	40	3 (8%)	<0.001
Pitting scars		-	397	160 (40%)		NA	NA
Rodnan skin score (median)	15	6 (5–11)	372	7 (4–12)		NA	NS
Renal crisis	25	1 (4%)	397	17 (4%)		NA	NS
***Myositis features***							
Serum CK elevation^‡^	25	24 (96%)	397	48 (12%)	40	34 (85%)	<0.001
Proximal muscle weakness	25	25 (100%)	397	21 (5%)	40	37 (93%)	<0.001
Myopathic EMG findings	25	22 (88%)		NA	29	24 (83%)	NS
Mechanic hands		NA		NA	40	7 (18%)	NS
***Internal complications***							
Arthritis	25	5 (20%)	397	46 (12%)	40	7 (18%)	NS
Interstitial lung disease							
HRCT fibrosis	18	15 (83%)	346	170 (49%)	19	10 (53%)	0.044
TLC ≤70% of predicted	21	4 (19%)	364	68 (19%)	23	9 (39%)	NS
TLCO ≤70% of predicted	17	17 (100%)	347	260 (75%)	20	13 (65%)	0.001
Diastolic dysfunction by cardiac ultrasound	19	7 (37%)	352	146 (42%)	19	4 (21%)	NS
PH suspicion by cardiac ultrasound	20	3 (15%)	352	89 (25%)	19	2 (11%)	0.042
PH by cardiac catherization	5	0	182	57 (31%)	2	1 (50%)	0.002
Myocarditis	25	1 (4%)		NA	40	0	NS
Malignancy	25	2 (8%)		NA	40	5 (13%)	NS

NA, not applicable; NS, not significant; TLC, total lung capacity; HRCT, high-resolution CT scan; TLCO, transfer factor of the lung for carbon monoxide; PH, pulmonary hypertension.

^‡^Defined >2 times upper limit of normal (ULN).

Signs of myositis such as serum CK elevation (>2 times ULN) and proximal muscle weakness were present in 48 SSc patients (12%) and in 21 SSc patients (5%), respectively (Table 1). Arthritis was present in all three groups, with a prevalence ranging from 12% to 20%.

### Interstitial lung disease

Interstitial lung disease (ILD) was diagnosed in patients whether or not moderate to severe pulmonary fibrosis was present on the HRCT scan. The presence of ILD was statistically significantly higher in the SSc-PM overlap group compared with the other two groups. Furthermore, a significantly higher prevalence of decreased lung carbon monoxide diffusion capacity (defined as TLCO ≤70%) was found in the SSc-PM overlap group (Table 2).

### Cardiac complications

Diastolic dysfunction on echocardiography was present in all three groups. Right heart catheterization (RHC) was performed only when pulmonary hypertension (PH) was suspected at echocardiography or in cases with unexplained dyspnea or an isolated decrease of carbon monoxide (CO) diffusion capacity at pulmonary-function testing. PH was diagnosed in none of the SSc-PM overlap patients, in 57 SSc patients, and in one PM patient. When no RHC was performed, we assumed that PH was absent. With this assumption, we found a statistically significant increase of PH in SSc patients compared with the other groups.

### Malignancy

In the SSc-PM overlap group, one patient developed a squamous cell carcinoma in the neck, and one patient developed a basal cell carcinoma of the skin on the forehead. In five patients in the PM group, a malignancy was diagnosed during follow-up, consisting of renal cell carcinoma (*n* = 1), melanoma (*n* = 1), breast cancer (*n* = 1), esophagus carcinoma (*n* = 1), and lung carcinoma (*n* = 1). Data concerning numbers and types of malignancy cases were not available in the SSc group.

### Mortality

Eight patients (32%) in the SSc-PM overlap group died, which was not statistically significantly increased compared with the other groups (Table 1). The causes of death were progressive heart disease (*n* = 2), myocardial infarction (*n* = 2) , respiratory failure due to interstitial lung disease (ILD) (*n* = 1) , sepsis (*n* = 2), and unknown cause (*n* = 1). Causes of mortality in the SSc group were progressive heart failure (*n* = 7) 10.8%, myocardial infarction (*n* = 2) 3.1%, respiratory failure due to ILD (*n* = 11) 16.9%, or pulmonary hypertension (*n* = 13) 20%, malignancies (*n* = 6) 9.2%, infections (*n* = 6) 9.2%, and other or unknown causes (*n* = 20) 30.8%. In the PM group, seven patients died. Their causes of death were progressive heart disease (*n* = 2), aspiration pneumonia (*n* = 2), malignancy (*n* = 2), and unknown cause (*n* = 1).

### Myopathology

**Figure 1:**
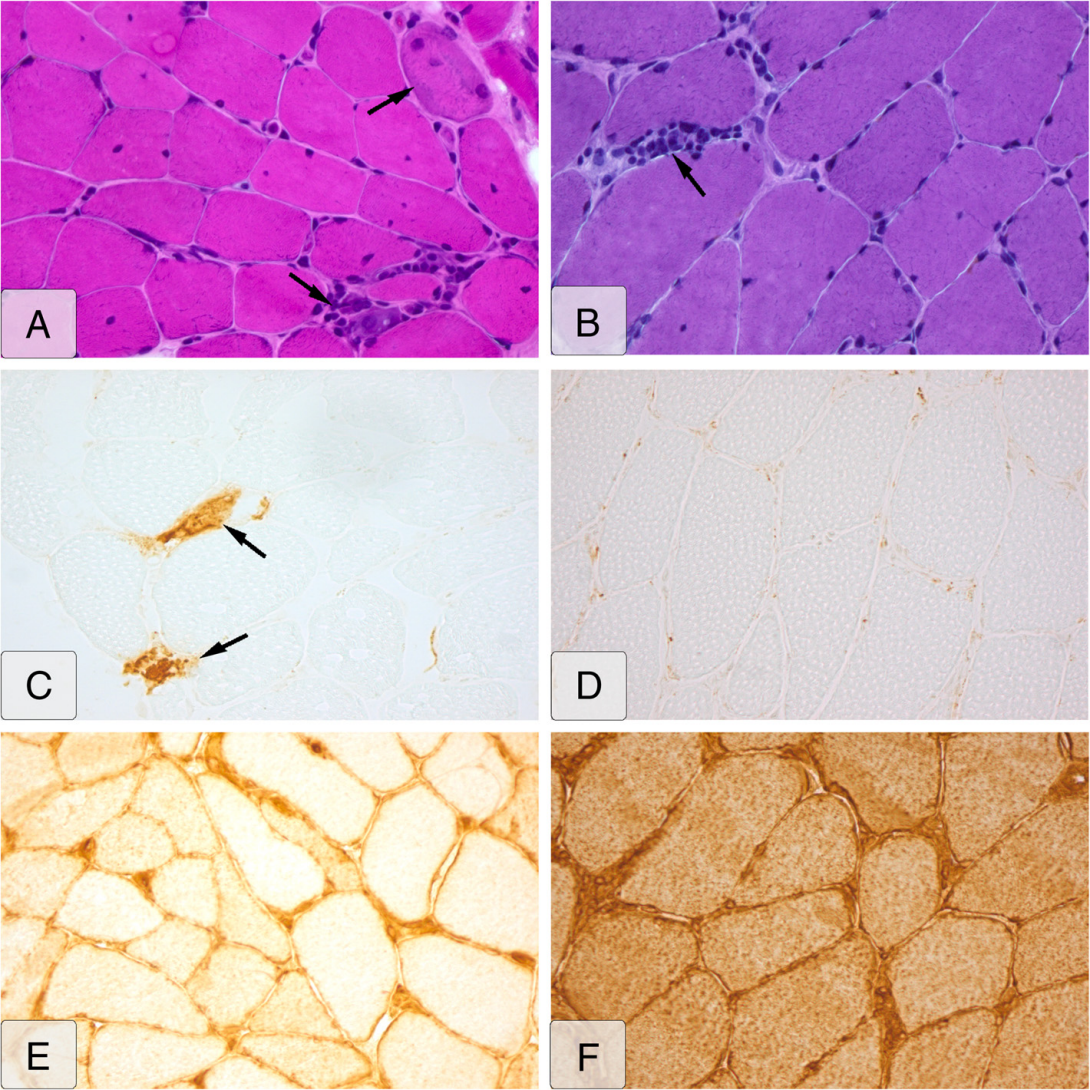
**Histopathologic muscle-biopsy images of SSc-PM overlap patient versus PM patient.** Left side **(A, C, E)**: SSc-overlap patient. Right side **(B, D, F)**: PM patient. **(A)** Hematoxylin & eosin (H&E) stain; upper marker, necrosis; lower marker, lymphocytic infiltrate; **(B)** H&E stain; marker, lymphocytic infiltrate; **(C)** MAC stain; markers, MAC upregulation; **(D)** MAC stain, no upregulation; (b v 02D7; **(E)** MHC class I stain, sarcolemmal MHC class I upregulation; **(F)** MHC class I stain, sarcolemmal and diffuse cytoplasmic MHC class I upregulation.

All muscle biopsies were assessed by the same neuropathologist who was blinded for the clinical diagnosis, and his findings are shown in Table 3 and Figure 1. We found a statistically significant difference in the presence of necrotic muscle fibers, present in 96% of the SSc-PM biopsies compared with 67% of the PM biopsies. This significant difference was caused mostly by differences in the presence of acute necrotic muscle fibers (50% versus 29.2%).

**Table 3:** Myopathologic markers of muscle biopsy slides

**Characteristics**	**Number**	**SSc-overlap (*n*** **= 24)**	**Number**	**PM (*n*** **= 24)**	**Significance (*P *** **value)**
Necrotic muscle fibers	24	23 (96%)	24	16 (67%)	0.023^b†^
Lymphocytic infiltrates	24	15 (63%)	24	13 (54%)	NS
Positive MHC class I staining	12	11 (92%)	18	12 (67%)	NS
Inflammation^a^	24	19 (79%)	24	18 (75%)	NS
MAC staining	10	5 (50%)	13	5 (39%)	NS
Fibrosis	19	5 (26%)	24	2 (8%)	NS
Presence of COX-negative fibers	10	3 (30%)	10	6 (60%)	NS

MAC, Membrane attack complex; NA, not applicable; NS, not significant.

^a^Inflammation is defined as positive MHC class I staining or/and presence of lymphocytic infiltrates.

^b^Statistically significant.

The presence of inflammatory markers such as lymphocytic infiltrates (63% versus 54%) and positive MHC I staining (92% versus 78%) were not significantly different among the SSc-PM overlap patients compared with the PM patients. Fibrosis displayed quite similar rates of presence in both groups. None of the muscle biopsies revealed rimmed vacuoles, ruling out inclusion-body myositis [21]. On characterization of different types of lymphocytes, only limited data were available. Deposition of MAC was found only in necrotic muscle fibers and not in the capillaries. COX and SDH staining was more often applied on the specimens and in a small number of cases (*n* = 3) in SSc-PM and (*n* = 6) in PM revealed the presence of COX-negative muscle fibers.

## Discussion

In this study, we describe and compare both the clinical characteristics and myopathologic findings of patients with SSc-PM overlap, SSc and PM. We found an increased prevalence of pulmonary fibrosis among patients with SSc-PM overlap, which is in accordance with a Japanese study that also showed an increased presence of ILD among patients with SSc-PM overlap compared with patients with SSc [7]. However, an increased presence of ILD was not confirmed in a French study [22]. ILD can cause early death and can diminish the quality of life because of symptoms of dyspnea and fatigue. However, no significant difference was found in ILD-associated death between the patients with SSc-PM overlap, SSc, and PM. However, our study has only a small study population, which limits a proper survival analysis with ILD. The increased prevalence of ILD in overlap patients should warrant regular screening for this complication and timely initiation of specific immunosuppressive therapy. Further studies should focus on the extent of pulmonary fibrosis and related morbidity among SSc-PM patients.

Several studies have described increased myocardial involvement, such as diastolic or systolic dysfunction and myocarditis, in patients with SSc-associated myopathies [7,9,22,23]. We found no statistically significant difference in myocardial involvement between the three patient cohorts in our study, but we used echocardiography as a screening tool, and cardiac MRI was not performed routinely. Echocardiography lacks specificity to diagnose myocarditis accurately, so the prevalence of this complication could prove to be higher if a cardiovascular MRI were performed as well [24]. In our cohort, we found progressive heart disease to be the cause of death in 25% of our overlap patients compared with only 10.8% of SSc patients.

The prevalence of SSc-PM overlap in our Nijmegen Systemic Sclerosis cohort was low (5.9%) compared with that in older investigations [3-7,9]. In more-recent studies, prevalences of 7.6% and 7.2% [22,25] were observed, which is comparable to our finding. This discrepancy in prevalences compared with older studies can be explained by the different criteria used to define SSc myopathy and the different study designs. In our study, we used strict criteria to select only inflammatory myopathies among SSc patients and not associated myopathies. Furthermore, this investigation includes only clinically relevant causes, whereas Medsger *et al*. [3] reported 90% prevalence of proximal muscle weakness, of which only 20% complained of muscle-related symptoms.

Different antibodies are associated with SSc-PM overlap, such as anti-PM/Scl and anti-Ku [26,27]. More-recent studies show novel antibodies related to SSc-PM, anti-U3 RNP, and anti-RuvBL1/2, respectively [28,29]. These antibodies relate to different clinical manifestations of SSc-PM-overlap patients; for example, Caucasian patients with limited cutaneous involvement and pulmonary fibrosis (antiPM/Scl) or African male patients with diffuse cutaneous involvement and pulmonary arterial hypertension (anti-U3 RNP) [26,28]. Unfortunately, no data concerning SSc-PM overlap-associated antibodies were available, which can be regarded as a limitation of our study.

We studied muscle biopsies of both overlap and PM patients, blinded for the diagnosis. For the first time, our study revealed that the myopathology in patients with SSc-PM overlap syndrome shows specific features compared with PM patients. Necrotizing muscle fibers were observed in almost all SSc-PM overlap samples. In the majority of the SSc-PM muscle biopsies, features of inflammation were also found, enabling us to exclude the histopathologic diagnosis of a necrotizing myopathy, according to the European Neuromuscular Centre workshop 2003 [30]. A French study showed a slightly lower prevalence of 25 (63%) of 35 necrotic muscle fibers among their cohort of SSc-PM overlap patients [8]. However, when including the presence of regenerating (that is, basophilic) muscle fibers, a feature present after necrosis, the prevalence of necrosis is in accordance with our findings.

MAC staining is often used to analyze muscle biopsies for signs of dermatomyositis (DM) if present as linear deposits at endothelial cells of endomysial capillaries [19,20]. This was not present in our study. We observed MAC staining in the sarcoplasm, which is indicative of necrotic fibers. No presence of rimmed vacuoles was found in this study. This verifies that our PM muscle biopsies are homogeneous for that diagnosis and are unlikely to include patients with IBM or DM.

The presence of COX-negative muscle fibers might be indicative of mitochondrial abnormalities. Growing evidence suggests that mitochondrial abnormalities can be found in dermatomyositis, inclusion-body myositis, and very seldom in polymyositis [21,31]. The results of our study revealed a small number of cases with COX-negative fibers. Only two PM patients had more than a single affected muscle fiber, but both patients were older than 65 years, and in these cases, the COX-negative fibers may be considered an effect of ageing. Therefore, we conclude that no evidence exists for mitochondrial pathology in patients with SSc-PM overlap.

This study has several limitations. All the subjects were recruited from a single, tertiary referral center, which may have led to a bias toward more-severe cases. However, this is true for all three patient cohorts, and therefore, it would less likely bias observed differences among the patient cohorts. The small sample size of the SSc-PM overlap cohort makes us cautious about overinterpretation of results. Nonetheless, by using strict criteria, we were able to identify a well-defined homogeneous overlap cohort. Furthermore, the blinded-for-diagnosis expert assessment of overlap muscle biopsies in comparison with PM biopsies is unique and greatly increased the strength of our findings.

## Conclusions

In conclusion, we found a prevalence of 5.9% of SSc-PM overlap in the Nijmegen Systemic Sclerosis cohort. Second, we observed an increased proportion of pulmonary fibrosis among patients with SSc-PM overlap. Third, we revealed that cardiac complications are a major cause of death in patients with SSc-PM overlap. Consequently, we urge more active and regular cardiopulmonary screening among these SSc-PM patients. Fourth, we found that necrotizing muscle fibers with inflammation characterize SSc-PM overlap in muscle biopsies. Further research should focus on underlying mechanisms causing necrosis, inflammation, and fibrosis and their relation to pulmonary involvement and mortality in SSc-PM overlap patients to optimize treatment, increase survival, and improve quality of life.

## Abbreviations

COX: Cytochrome C-oxidase; EMG: electromyography; HRCT scan: high-resolution computed tomography scan; ILD: interstitial lung disease; MAC: membrane attack complex; mRSS: modified Rodnan skin score; PM: polymyositis; SDH: succinate dehydrogenase; SSc: systemic sclerosis; SSc-PM: scleroderma-polymyositis.

## Competing interests

All authors declare not to have received any financial support or other benefits from commercial sources for work reported here or to have any interests that could create a potential conflict of interest or appearance of conflict of interest with regard to this work.

## Authors’ contributions

KB participated in the design of the study and carried out data collection, statistical analysis, and drafting of the manuscript. ML carried out evaluation of the muscle biopsies and participated in the design and drafting of the manuscript. HK was involved in the design of the study, and helped in the coordination and drafting of the manuscript. PvR participated in the design of the study, interpretation of the data, drafting, and critical revising of the manuscript. BvE participated in the design of the study, interpretation of the data, and critical revising of the manuscript. MV conceived the study, and participated in the coordination, interpretation of the data, and drafting of the manuscript. All authors read and approved the final manuscript.

## References

[B1] Furst DE Clements PJ Hypothesis for the pathogenesis of systemic sclerosis J Rheumatol Suppl 1997 48 53 57 9150119

[B2] Medsger TA Jr Masi AT Epidemiology of systemic sclerosis (scleroderma) Ann Intern Med 1971 74 714 721 10.7326/0003-4819-74-5-714 5559436

[B3] Medsger TA Jr Rodnan GP Moossy J Vester JW Skeletal muscle involvement in progressive systemic sclerosis (scleroderma) Arthritis Rheum 1968 11 554 568 10.1002/art.1780110405 5676926

[B4] Thompson JM Bluestone R Bywaters EG Dorling J Johnson M Skeletal muscle involvement in systemic sclerosis Ann Rheum Dis 1969 28 281 288 10.1136/ard.28.3.281 18623879 PMC1031178

[B5] Tuffanelli DL Scleroderma and its relationship to the “collagenoses”: dermatomyositis, lupus erythematosus, rheumatoid arthritis and Sjogren’s syndrome Am J Med Sci 1962 243 133 146 13923027

[B6] Clements PJ Furst DE Campion DS Bohan A Harris R Levy J Paulus HE Muscle disease in progressive systemic sclerosis: diagnostic and therapeutic considerations Arthritis Rheum 1978 21 62 71 10.1002/art.1780210111 623695

[B7] Mimura Y Ihn H Jinnin M Asano Y Yamane K Tamaki K Clinical and laboratory features of scleroderma patients developing skeletal myopathy Clin Rheumatol 2005 24 99 102 10.1007/s10067-004-0975-7 15322944

[B8] Ranque B Authier FJ Le-Guern V Pagnoux C Berezne A Allanore Y Launay D Hachulla E Kahan A Cabane J Gherardi R Guillevin L Mouthon L A descriptive and prognostic study of systemic sclerosis-associated myopathies Ann Rheum Dis 2008 68 1474 1477 19054827 10.1136/ard.2008.095919

[B9] Follansbee WP Zerbe TR Medsger TA Jr Cardiac and skeletal muscle disease in systemic sclerosis (scleroderma): a high risk association Am Heart J 1993 125 194 203 10.1016/0002-8703(93)90075-K 8417518

[B10] Tuffanelli DL Winkelmann RK Systemic scleroderma: a clinical study of 727 cases Arch Dermatol 1961 84 359 371 10.1001/archderm.1961.01580150005001 13778561

[B11] Mimori T Scleroderma-polymyositis overlap syndrome: clinical and serologic aspects Int J Dermatol 1987 26 419 425 10.1111/j.1365-4362.1987.tb00580.x 3308722

[B12] Hashimoto A Tejima S Tono T Suzuki M Tanaka S Matsui T Tohma S Endo H Hirohata S Predictors of survival and causes of death in Japanese patients with systemic sclerosis J Rheumatol 2011 38 1931 1939 10.3899/jrheum.100298 21765111

[B13] van Engelen BG van Veenendaal H van Doorn PA Faber CG van der Hoeven JH Janssen NG Notermans NC van Schaik IN Visser LH Verschuuren JJ The Dutch neuromuscular database CRAMP (Computer Registry of All Myopathies and Polyneuropathies): development and preliminary data Neuromusc Disord 2007 17 33 37 10.1016/j.nmd.2006.09.017 17141501

[B14] Subcommittee for scleroderma criteria of the American Rheumatism Association Diagnostic and Therapeutic Criteria Committee Preliminary criteria for the classification of systemic sclerosis (scleroderma) Arthritis Rheumatism 1980 23 581 590 10.1002/art.1780230510 7378088

[B15] LeRoy EC Medsger TA Jr Criteria for the classification of early systemic sclerosis J Rheumatol 2001 28 1573 1576 11469464

[B16] Bohan A Peter JB Polymyositis and dermatomyositis (first of two parts) N Engl J Med 1975 292 344 347 10.1056/NEJM197502132920706 1090839

[B17] Bohan A Peter JB Polymyositis and dermatomyositis (second of two parts) N Engl J Med 1975 292 403 407 10.1056/NEJM197502202920807 1089199

[B18] van der Pas J Hengstman GJ Ter Laak HJ Borm GF van Engelen BG Diagnostic value of MHC class I staining in idiopathic inflammatory myopathies J Neurol Neurosurg Psychiatry 2004 75 136 139 14707323 PMC1757482

[B19] Kissel JT Mendell JR Rammohan KW Microvascular deposition of complement membrane attack complex in dermatomyositis N Engl J Med 1986 314 329 334 10.1056/NEJM198602063140601 3945256

[B20] Jain A Sharma MC Sarkar C Bhatia R Singh S Gulati S Handa R Detection of the membrane attack complex as a diagnostic tool in dermatomyositis Acta Neurol Scand 2011 123 122 129 10.1111/j.1600-0404.2010.01353.x 20497129

[B21] Dalakas MC Sporadic inclusion body myositis: diagnosis, pathogenesis and therapeutic strategies Nat Clin Pract Neurol 2006 2 437 447 10.1038/ncpneuro0261 16932602

[B22] Ranque B Berezne A Le-Guern V Pagnoux C Allanore Y Launay D Hachulla E Authier FJ Gherardi R Kahan A Cabane J Guillevin L Mouthon L Myopathies related to systemic sclerosis: a case–control study of associated clinical and immunological features Scand J Rheumatol 2010 39 498 505 10.3109/03009741003774626 20726682

[B23] West SG Killian PJ Lawless OJ Association of myositis and myocarditis in progressive systemic sclerosis Arthritis Rheum 1981 24 662 668 10.1002/art.1780240506 7236323

[B24] Friedrich MG Sechtem U Schulz-Menger J Holmvang G Alakija P Cooper LT White JA Abdel-Aty H Gutberlet M Prasad S Aletras A Laissay JP Paterson I Filipchuk NG Kumar A Pauschinger M Liu P Cardiovascular magnetic resonance in myocarditis: a JACC White Paper J Am Coll Cardiol 2009 53 1475 1487 10.1016/j.jacc.2009.02.007 19389557 PMC2743893

[B25] Tyndall AJ Bannert B Vonk M Airo P Cozzi F Carreira PE Bancel DF Allanore Y Müller-Ladner U Distler O Iannone F Pellerito R Pileckyte M Miniati I Ananieva L Gurman AB Damjanov N Mueller A Valentini G Riemekasten G Tikly M Hummers L Henriques MJ Caramaschi P Scheja A Rozman B Ton E Kumánovics G Coleiro B Feierl E Causes and risk factors for death in systemic sclerosis: a study from the EULAR Scleroderma Trials and Research (EUSTAR) database Ann Rheum Dis 2010 69 1809 1815 10.1136/ard.2009.114264 20551155

[B26] Oddis CV Okano Y Rudert WA Trucco M Duquesnoy RJ Medsger TA Jr Serum autoantibody to the nucleolar antigen PM-Scl: Clinical and immunogenetic associations Arthritis Rheum 1992 35 1211 1217 10.1002/art.1780351014 1418007

[B27] Mimori T Akizuki M Yamagata H Inada S Yoshida S Homma M Characterization of a high molecular weight acidic nuclear protein recognized by autoantibodies in sera from patients with polymyositis-scleroderma overlap J Clin Invest 1981 68 611 620 10.1172/JCI110295 7276162 PMC370841

[B28] Aggarwal R Lucas M Fertig N Oddis CV Medsger TA Jr Anti-U3 RNP autoantibodies in systemic sclerosis Arthritis Rheum 2009 60 1112 1118 10.1002/art.24409 19333934

[B29] Kaji K Fertig N Medsger TA Jr Satoh T Hoshino K Hamaguchi Y Hasewaga M Lucas M Schnure A Ogawa F Sato S Takehara K Fuijmoto M Kuwana M Autoantibodies to RuvBL1 and RuvBL2: a novel systemic sclerosis-related antibody associated with diffuse cutaneous and skeletal muscle involvement Arthritis Care Res (Hoboken) 2014 66 575 584 10.1002/acr.22163 24023044

[B30] Hoogendijk JE Amato AA Lecky BR Choy EH Lundberg IE Rose MR Vencovsky J de Visser M Hughes RA 119th ENMC international workshop: trial design in adult idiopathic inflammatory myopathies, with the exception of inclusion body myositis, 10–12 October 2003, Naarden, The Netherlands Neuromuscul Disord 2004 14 337 345 10.1016/j.nmd.2004.02.006 15099594

[B31] Varadhachary AS Weihl CC Pestronk A Mitochondrial pathology in immune and inflammatory myopathies Curr Opin Rheumatol 2010 22 651 657 10.1097/BOR.0b013e32833f108a 20827203

